# Back to the roots, desiccation and radiation resistances are ancestral characters in bdelloid rotifers

**DOI:** 10.1186/s12915-023-01554-w

**Published:** 2023-04-07

**Authors:** Boris Hespeels, Diego Fontaneto, Valérie Cornet, Sébastien Penninckx, Jérémy Berthe, Lucie Bruneau, James W. Larrick, Eloïse Rapport, Jérémie Bailly, Nicolas Debortoli, Nataliia Iakovenko, Karel Janko, Anne-Catherine Heuskin, Stéphane Lucas, Bernard Hallet, Karine Van Doninck

**Affiliations:** 1 Research Unit in Environmental and Evolutionary Biology (URBE), Laboratory of Evolutionary Genetics and Ecology (LEGE), NAmur Research Institute for Life Sciences (NARILIS), University of Namur, Namur, Belgium; 2 Research Unit in Environmental and Evolutionary Biology (URBE), Institute of Life, Earth & Environment (ILEE), University of Namur, Namur, Belgium; 3 Molecular Ecology Group (MEG), Water Research Institute (IRSA), National Research Council of Italy (CNR), Verbania Pallanza, Italy; 4 Laboratory of Non-Mendelian Evolution, Institute of Animal Physiology and Genetics AS CR, Rumburská 89, Liběchov, 277 21 Czech Republic; 5 Medical Physics Department, Institut Jules Bordet, Université Libre de Bruxelles, Brussels, Belgium; 6 Research Unit in Molecular Biology and Evolution, DBO, Université libre de Bruxelles (ULB), 1050 Brussels, Belgium; 7 Panorama Research Institute, Sunnyvale, CA USA; 8 SETI Institute, Mountain View, CA USA; 9 Faculty of Forestry and Wood Sciences, Czech University of Life Sciences Prague, Kamýcká 129, CZ - 165 21 Praha 6, Suchdol, Czech Republic; 10 Faculty of Science, University of Ostrava, Chittussiho 10, 71000 Ostrava, Czech Republic; 11 Laboratory of Analysis by Nuclear Reactions (LARN), Namur Research Institute for Life Sciences (Narilis), University of Namur, Namur, Belgium; 12 Louvain Institute of Biomolecular Science and Technology, UCLouvain, B-1348 Louvain-la-Neuve, Belgium

**Keywords:** Desiccation, DNA repair, Extreme tolerance, Bdelloid rotifers, Ancestral character reconstructions, Atacama Desert, Antarctica

## Abstract

**Background:**

Bdelloid rotifers are micro-invertebrates distributed worldwide, from temperate latitudes to the most extreme areas of the planet like Antarctica or the Atacama Desert. They have colonized any habitat where liquid water is temporarily available, including terrestrial environments such as soils, mosses, and lichens, tolerating desiccation and other types of stress such as high doses of ionizing radiation (IR). It was hypothesized that bdelloid desiccation and radiation resistance may be attributed to their potential ability to repair DNA double-strand breaks (DSBs). Here, these properties are investigated and compared among nine bdelloid species collected from both mild and harsh habitats, addressing the correlation between the ability of bdelloid rotifers to survive desiccation and their capacity to repair massive DNA breakage in a phylogenetically explicit context. Our research includes both specimens isolated from habitats that experience frequent desiccation (at least 1 time per generation), and individuals sampled from habitats that rarely or never experienced desiccation.

**Results:**

Our analysis reveals that DNA repair prevails in somatic cells of both desiccation-tolerant and desiccation-sensitive bdelloid species after exposure to X-ray radiation. Species belonging to both categories are able to withstand high doses of ionizing radiation, up to 1000 Gy, without experiencing any negative effects on their survival. However, the fertility of two desiccation-sensitive species, *Rotaria macrura* and *Rotaria rotatoria*, was more severely impacted by low doses of radiation than that of desiccation-resistant species. Surprisingly, the radioresistance of desiccation-resistant species is not related to features of their original habitat. Indeed, bdelloids isolated from Atacama Desert or Antarctica were not characterized by a higher radioresistance than species found in more temperate environments.

**Conclusions:**

Tolerance to desiccation and radiation are supported as ancestral features of bdelloid rotifers, with a group of species of the genus Rotaria having lost this trait after colonizing permanent water habitats. Together, our results provide a comprehensive overview of the evolution of desiccation and radiation resistance among bdelloid rotifers.

**Supplementary Information:**

The online version contains supplementary material available at 10.1186/s12915-023-01554-w.

## Background

Bdelloid rotifers are among the smallest animals on Earth, with most species comprised of ~1000 cells, and being less than half millimeters in length. These eutelic (i.e., containing a fixed number of cells at maturity) metazoans harbor clearly differentiated nervous, muscular, digestive, excretory, and reproductive systems [1]. A remarkable feature of bdelloid rotifers, other than the absence of males in any of the 460 described species [2–4], is their worldwide occurrence, including environments that are considered among the most extreme ones on Earth, like those found in Antarctica “coldest” or Atacama “driest and highest UV irradiation” deserts [5, 6]. Very few bdelloids live in permanently hydrated conditions, with more than 95% of the species inhabiting limno-terrestrial environments (i.e., terrestrial habitats where liquid water is temporarily available) such as soils, mosses, and lichens, or in ephemeral water bodies [7–9]. In these environments, temperature, food accessibility, chemical composition, water availability, and other variables can change quickly and unpredictably [10]. Bdelloids are able to colonize such unpredictable habitats thanks to an unprecedented capacity to survive long periods of drought by entering a metabolically quiescent state of anhydrobiosis (also called desiccation) at any stage of their life cycle. Upon rehydration, they are able to resume activity and reproduction and do not appear to be negatively affected by such periods of desiccation [11]. Notably, several studies have reported that the fitness of specific bdelloid rotifer species declines under permanently hydrated conditions when compared to lines that were exposed to repeated cycles of desiccation [10–12].

However, few bdelloid species only dwell permanently in water where they apparently never experience desiccation. For example, *Rotaria macrura* is found only in permanent ponds and does not exhibit any tolerance to desiccation [8, 13, 14]. This appears to be also the case for other species of the same genus, e.g., *Rotaria magnacalcarata* and *Rotaria socialis*, living as epibionts (i.e., an organism living on the surface of another organism) on the freshwater crustacean *Asellus aquaticus* [1, 13, 15].

Tolerance to desiccation of limno-terrestrial bdelloids appears to be responsible for their resistance to a variety of environmental stressors including high pressure, vacuum, and freezing [16–20]. In particular, desiccation-resistant bdelloid rotifers were also shown to have a high tolerance to ionizing radiation (i.e., particles or electromagnetic waves that have sufficient energy to ionize atoms or molecules). Gladyshev and Meselson [17, 21] reported that exposure of hydrated *Adineta vaga* and *Philodina roseola* individuals to high doses of γ-radiation, ranging from 280 to 560 Gy, caused hundreds of DNA double-strand breaks (DSB) per genome without affecting their survival or reproduction. By analogy with the desiccation- and radiation-resistant bacterium *Deinococcus radiodurans*, radiation resistance in bdelloid rotifers has been hypothesized to reflect their capacity to survive desiccation in semi-terrestrial habitats [17, 22, 23]. Consistent with this hypothesis, prolonged periods of desiccation were shown to induce substantial amounts of DNA DSBs in the bdelloid rotifer *A. vaga*, which, like those produced by X-ray, proton, and Fe radiations, were efficiently repaired upon rehydration [19, 20].

Desiccation-induced DNA DSBs have been hypothesized to act as a gateway for genetic exchange in bdelloid rotifers [13, 17, 19, 23]. Upon desiccation, the integrity of cell membranes is compromised and DNA breaks accumulate, which could promote the uptake and integration of foreign DNA during the repair process [13, 17, 19, 21, 24, 25]. This hypothesis was initially proposed based on primary genomic and transcriptomic data obtained for *A. ricciae* and *A. vaga* [24]*,* showing that both desiccation-resistant species contained around 8 to 10% of horizontally acquired non-metazoan genes. However, a more recent comparative study revealed no significant difference in terms of horizontal gene transfer (HGT) frequency between frequently desiccating bdelloid species and species living in permanent water bodies [14], making the hypothesized process not grounded on solid proof and requiring more experimental evidence. Finally, a larger study focusing on 24 desiccation-resistant eukaryotic species (including bdelloid, tardigrade, nematode, insect, fungi, and plant species) was not able to highlight a significant higher proportion of exogenous DNA captures in these species when compared to desiccation-sensitive species [26].

**Fig. 1:**
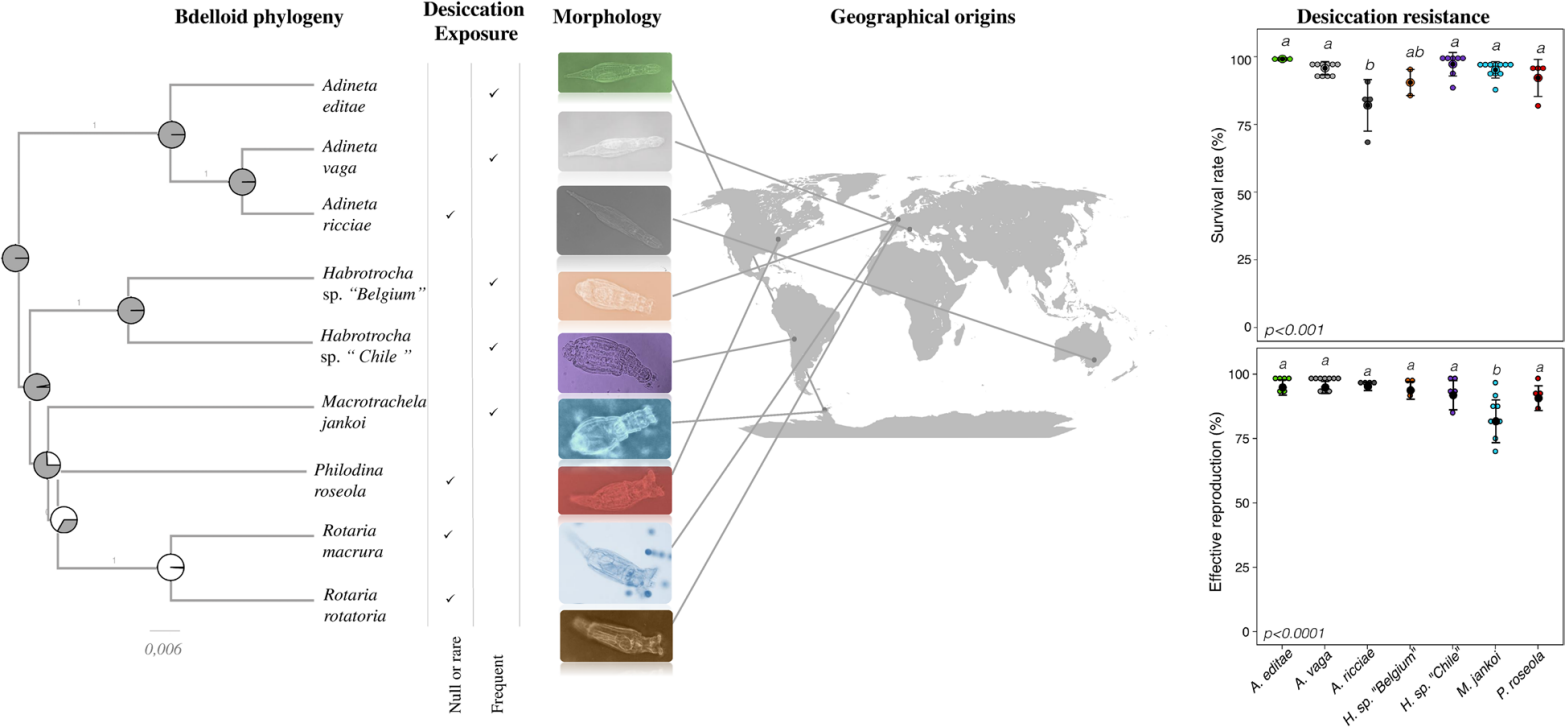
Phylogeny, desiccation features, geographical origin and desiccation resistance of bdelloid species exposed to desiccation (with the exception of desiccation-sensitive *R. macrura* and *R. rotatoria* species). Phylogeny (Left) was reconstructed from Cox1 and 18S. The tree was calibrated using published substitution rates of Cox1 and 18S (see Methods). All clades have posterior probability support values = 1.0. *Adineta vaga* species was added as reference species with published genomic data (Flot et al. [27]). Mapped ancestral character reconstruction (ACE) was added on the phylogenetic tree for desiccation resistance. Color codes are white = desiccation-sensitive and gray = desiccation-tolerant. Bdelloids were classified in 2 groups associated with their exposure to desiccation: Group “Frequent” includes species isolated from habitats that experience frequent desiccation (at least 1 time per generation); Group “Null or rare” includes species sampled from habitats that rarely or never experienced desiccation (see Methods). Map (Center) was acquired from https://upload.wikimedia.org/wikipedia/commons/6/69/World_map_blank_gmt.svg. Illustration pictures credited to B. Hespeels and D. Fontaneto. Except for the two *Rotaria* species, all species were found to be desiccation tolerant. Comparison between the survival rate and the capacity to produce a fertile population of desiccation-resistant bdelloid species exposed to 24h of desiccation (Right) was visualized as a dotplot (● = average value with plot of Standard Deviation). Survival rate was evaluated 2 days post rehydration on a minimum of 3 replicates. Fertility rate was evaluated 1 month post isolation of rehydrated individuals on a minimum of 3 replicates (see Methods). For each replicate, 60 individuals were randomly isolated and individually placed in multi-well plates. Reproduction was evaluated by direct observation under a binocular stereoscope 30 days after rehydration/radiation. Effective reproduction was validated when at least 2 adults and 1 egg were observed per well. Letters indicate significant differences between groups: a significant difference (Tukey test *p*-value <0.05) between two conditions is observed when these conditions do not share any letter. Desiccation resistance data of *A. vaga* species was extracted from Hespeels et al. [20]

In this paper, we investigate the correlation between the ability of bdelloid rotifers to survive desiccation and reproduce, and their capacity to repair massive DNA breakage. In particular, we sought to determine whether tolerance to desiccation is an ancestral characteristic of bdelloid rotifers, with only a few species having “recently (or secondary)” lost this aptitude when they colonized permanent waterbodies or if tolerance to desiccation was acquired when colonizing limno-terrestrial habitats, and whether desiccation-sensitive bdelloid species are still able to survive high doses of ionizing radiations and to repair high levels of DNA DSBs. Indeed, it remains unclear whether tolerance to DNA DSBs can be extended to all bdelloid species or not. So far, the recovery of genomic integrity post desiccation and/or irradiation was only investigated in the model species *A. vaga* [19, 20, 23]. Finally, we determined if radioresistance, expressed at the reproductive fitness, is different between species with the expectation that species living in extreme habitats would have better resistance. The study was performed by comparing nine bdelloid species from different families that were collected in different areas of the world (Antarctica, Belgium, Chile, Italy, and USA) and living in distinct habitats (polar areas, deserts, lichens, mosses, permanent and temporary ponds, soils) (See Fig. 1). Survival and fertility of the different species were compared under both stress conditions (desiccation and radiation) and by analyzing the subsequent repair of DNA DSBs.

## Results

### Desiccation and radiation survival

**Fig. 2:**
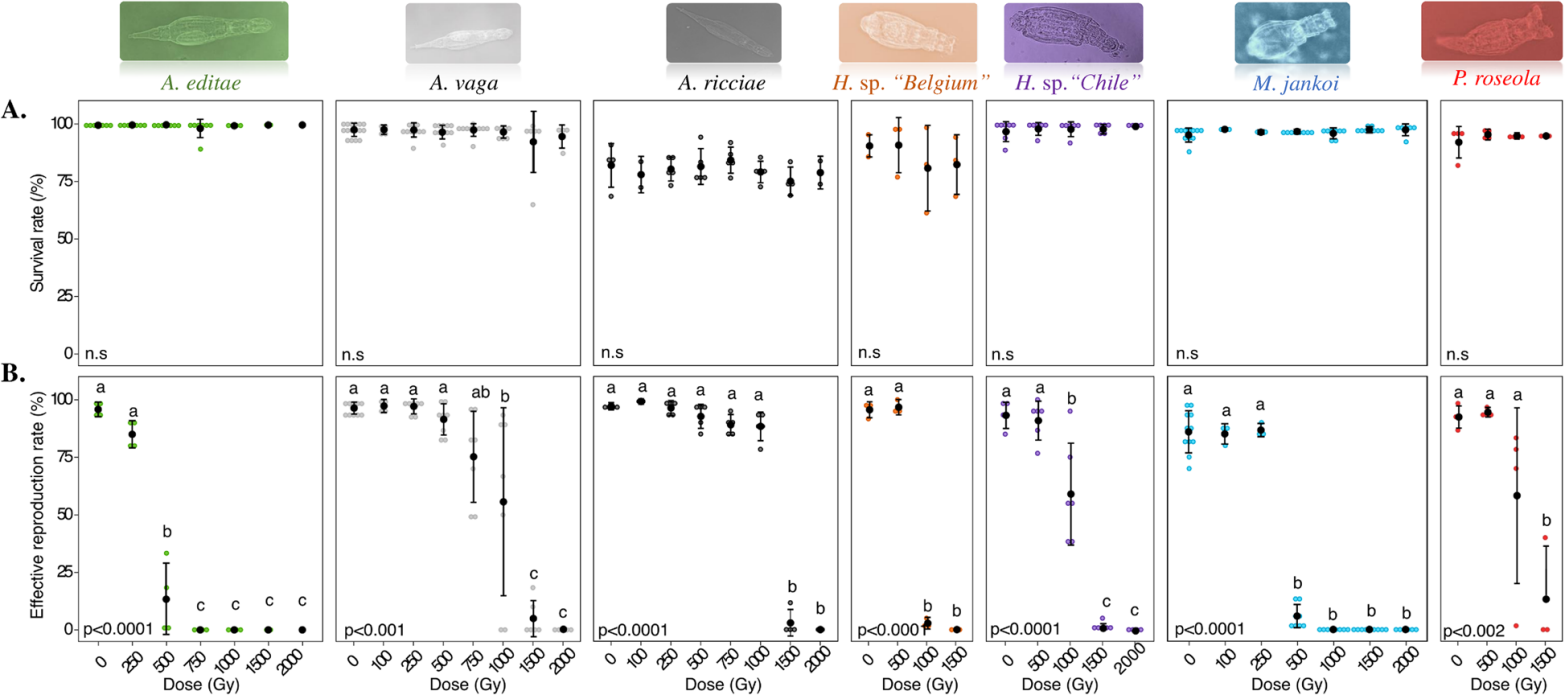
Survival rate (**A**) and reproductive capacity (**B**) of desiccation-tolerant bdelloid rotifers (respectively, *Habrotocha* sp. *“Chile”*, *A. editae*, *A. ricciae*, *P. roseola*, *Habrotrocha* sp. *“Belgium”* and *M. jankoi*) exposed to desiccation and increasing doses of X-ray. Survival rate of 1-day desiccated and irradiated bdelloid individuals was evaluated 48h post rehydration. 0 Gy correspond to 24-h desiccated bdelloids. Statistical interpretation of the interaction between survival rate and radiation dose is reported at the bottom of each graph. The reproductive capacity was calculated with a minimum of 3 replicates per dose (see Methods). For each replicate, 60 individuals were randomly isolated and individually placed in multi-well plates. Reproduction was evaluated by direct observation under binocular stereoscope 30 days after rehydration/radiation. Effective reproduction was validated when at least 2 adults and 1 egg were observed per well. Data were visualized as a dotplot (● = average value with plot of standard deviation). *P* values (P) of GLM test are reported on each graph. Letters indicate significant differences between groups: a significant difference (Tukey test *p*-value <0.05) between two conditions is observed when these conditions do not share any letter

For the seven desiccation-tolerant species, the proportion of animals surviving 24-h desiccation was relatively high as expected, ranging from ~75 to ~100% (Figs. 1 and 2, Additional file 2). Survival rates of Antarctic species (*A. editae* (99.1±0.4%) and *M. jankoi* (95±3%)), species originating from the Atacama Desert (*Habrotrocha* sp. *“Chile”* (97,1±4.3*%))*, and two other species, *P. roseola* (92.1±6.9%) and *Habrotrocha* sp*. “Belgium”* (89.7±4.8%), living in temperate habitats were not significantly different. However, the survival rates of the other desiccation-tolerant species, *A. ricciae* (81.6±9.5%), were significantly lower than those measured for most of the other species (Fig. 1).

Previously, it had been shown that 1-day desiccation did not affect the fertility of *A. vaga* individuals. Here, the capacity of 1-day desiccated animals to produce fertile eggs after rehydration was high (>90%) and was not different between tested species with the exception of *M. jankoi* (effective reproduction rate: 83.6±11.1%) (Fig. 1)*.* For this species, we hypothesized a laboratory effect with unidentified parameters such as temperatures or presence of uncharacterized fungi/bacteria (originated from the original habitats (i.e., geographical site where the bdelloid individual was sampled in the field)) may have impacted the fertility assay. For the two desiccation-sensitive species *R. macrura* or *R. rotatoria*, no animal survived desiccation, consistent with the previously reported desiccation-sensitive status of these species [14].

In spite of their good aptitude to survive under dehydrated conditions, pulsed-field gel electrophoresis (PFGE) analysis of the three desiccation-tolerant bdelloid species that were selected for a more detailed experiment (i.e., *P. roseola, Habrotrocha* sp. *“Belgium”* and *A. editae*) showed that DNA DSBs accumulated upon desiccation as was previously reported for the model species *A. vaga* [19] (Additional file 1: Fig. S1). For two species, *P. roseola* and *Habrotrocha* sp. *“Belgium”*, the amount of chromosomal DNA that entered the gel to form a band at the limit of gel resolution (i.e., 2200 kb) and a smear of smaller fragments (from 225 to 1600 kb) when exposed to 14 days and 1 month of desiccation was higher than in control or 1-day desiccated individuals, respectively. PFGE signal was, however, weaker for *A. editae* (also in the hydrated specimens). However, the amount of high molecular weight fragments (i.e., 2200 kb) was higher after 14 days and 1 month of desiccation exposure and a faint smear was visible demonstrating accumulation of DNA DSBs compared with the control and early desiccated stage samples. These results show that the ability to survive prolonged desiccation does not correlate with a capacity to prevent DNA DSB damages inherent to dehydration.

Survival to desiccation was found to have a phylogenetic influence for the nine species included in the study (Pagel’s lambda = 1.2, *p* = 0.0251; Blomberg’s *K* = 1.4, *p* = 0.0600; Fig. 1 and Additional file 1: Fig. S2). The ancestral character estimation suggests that the common ancestor of all the analyzed species already had the ability to resist to desiccation (scaled likelihood = 0.99) and to survive radiation (0.99). The estimation is however more ambiguous as to whether the ancestral habitat was rarely desiccating (0.49) or frequently experiencing desiccation (0.51) (Additional file 1: Fig. S2). After controlling for phylogenetic relatedness, survival significantly depended on the frequency of desiccation of the habitat where the species lives (rarely vs frequently desiccating, PGLS: *t* = 2.8, *p* = 0.0277, adjusted *R*^2^ = 0.45), with higher survival among species coming from habitats experiencing frequent desiccation.

**Fig. 3:**
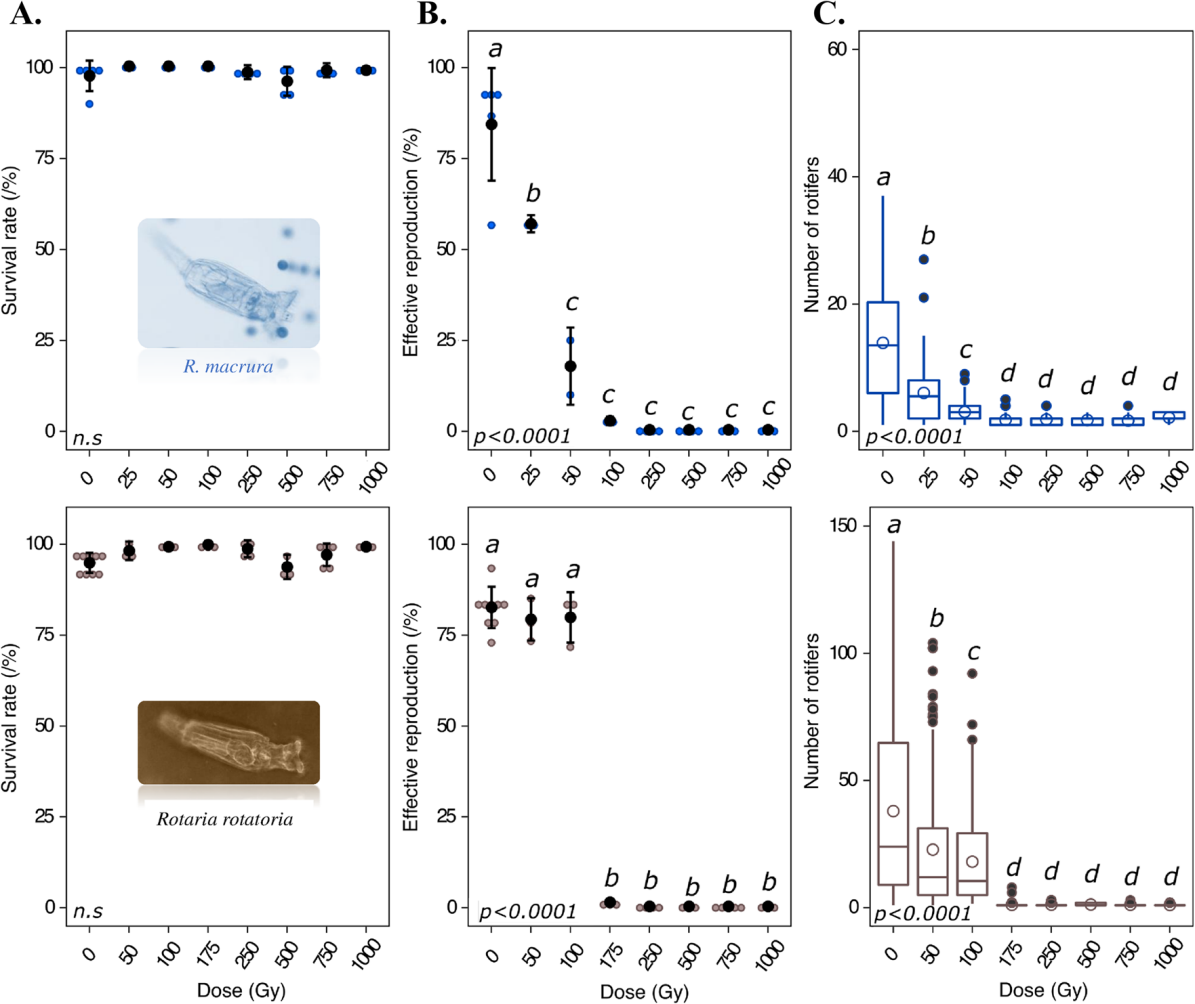
Survival rate (**A**) and reproductive capacity (**B, C**) of *Rotaria macrura* and *Rotaria rotatoria* individuals exposed to increasing doses of X-ray (up to 1000 Gy). Survival rate of hydrated and irradiated *R. macrura*/*R. rotatoria* individuals was evaluated 48h post isolation/radiation exposure (see Methods). For each replicate, 60 individuals were randomly isolated and individually placed in multi-well plates. Reproduction was evaluated by direct observation under binocular stereoscope 30 days after radiation. Effective reproduction, defined as the capacity to restart a culture from a single individual, was validated when at least 5 adults per well were observed 1 month post isolation (data were scaled to the 60 isolated individuals). Fecundity was also evaluated by counting the number of living or dead individuals 1 month after the isolation of a single individual in a single well. Data were visualized as a dotplot (● = average value with plot of standard deviation). *P* values (*P*) of GLM test are reported on each graph. Letters indicate significant differences between groups: a significant difference (Tukey test *p*-value <0.05) between two conditions is observed when these conditions do not share any letter

The seven desiccation-resistant bdelloid species (all except the two *Rotaria*) tolerated increasing doses of X-ray radiation (up to 1500 Gy and 2000 Gy) with no impact on survival rate (Fig. 2A). Similarly, no decrease in survival (48 h post irradiation) was detected for the desiccation-sensitive *R. macrura* and *R. rotatoria* exposed to up to 1000 Gy of X-rays while hydrated (Fig. 3). Thus, both the desiccation-tolerant and desiccation-sensitive species appear to survive high doses of radiation.

The percentages of radiation survival measured at 500, 1000, and 1500 Gy for the seven desiccation-tolerant species exposed while desiccated were highly correlated (Pearson’s *r* = 0.93 to 0.97, *t* = 5.6 to 8.5, *p* = 0.0004 to 0.0026). Thus, only one of these results (arbitrarily chosen at 1000 Gy) will be used in the following statistical analyses as a proxy for survival after radiation. Survival capability for the seven desiccation-tolerant species after exposure to radiation (1000 Gy) had no phylogenetic signal (Pagel’s lambda = 0.1, *p* = 0.9998; Blomberg’s *K* = 0.5, *p* = 0.8759) but was positively explained by the degree of survival to desiccation of each species (PGLS: *t* = 17.3, *p* < 0.0001, adjusted R^2^ = 0.98).

### Reproductive capacity after exposure to radiation

The fertility rates measured for the different bdelloid species at different doses of X-ray irradiation show that their ability to produce viable progeny is more sensitive to ionizing radiations than their simple survival (Figs. 2 and 3).

The desiccation-sensitive species *R. macrura* was sterilized by 100 Gy of irradiation and above (Fig. 3B, C) being unable to produce populations with more than 4 individuals 30 days post irradiation. This number reflects the capacity for embryos that were irradiated inside their viviparous mother to complete their maturation and to be released in the environment without being able to produce a viable progeny (Fig. 3C). A significant drop of the fertility was already observed after exposure to 25 Gy of X-rays, which was correlated with a marked reduction in *R. macrura* population (Fig. 3B). For the second desiccation-sensitive species *R. rotatoria*, most individuals were unable to produce any offspring or maximally one sterile individual at a dose of 175 Gy and above (Fig. 3B, C). As observed for *R. macrura*, a unique juvenile laid by the irradiated mother likely reflects the capacity of the single embryo to complete its maturation post irradiation. Only 2/180 isolated animals were able to produce more than 5 individuals 1-month post irradiation after an irradiation to 175 Gy. No fertile individuals were observed for higher doses (Fig. 3B).

**Fig. 4:**
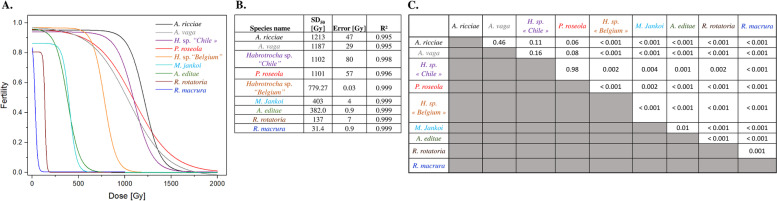
Comparison of radiation Sterilizing Dose 50 (SD_50_) between different species of bdelloid rotifers. A similar approach to the evaluation of a standard LD50 was applied here to define SD_50_ of each bdelloid species. SD_50_ represents the minimal dose required to sterilize 50% of irradiated population. All curve fittings were performed with the OriginLab® software (MA, USA)

The reproductive capacity of irradiated bdelloids was compared between the different bdelloid species based on the minimal dose required to sterilize 50% of the population (SD_50_) (Fig. 4 and Additional file 1: Fig. S3). The lowest median doses to sterilize a population (Sterilizing Dose 50 SD_50_) were obtained for the desiccation-sensitive species *R. macrura* (31 ± 1 Gy) and *R. rotatoria* (137 ± 7 Gy). These values are significantly lower than the SD_50_ values measured for the desiccation-resistant species, ranging from 382 ± 1 Gy and 403 ± 4 Gy for the Antarctic species *A. editae* and *M. jankoi* to ~1100–1200 Gy for the most resistant species (*P. roseola* 1,101 ± 57 Gy; *Habrotrocha* sp. “*Chile”* 1,102 ± 81 Gy and *A. ricciae* 1,213 ± 47 Gy) (Fig. 4 A–C). The latter values are in the same range as the SD_50_ reported for *A. vaga* (1187 ± 29 Gy, Hespeels et al. [20]), suggesting that these values may correspond to a standard maximum level of resistance among bdelloid rotifers coming from different environments.

In congruence with these data, no significant decrease in fertility was reported up to 500 Gy for *Habrotrocha* sp. *“Chile”*. Similarly, fertility was not affected up to 750 Gy for *A. vaga,* and 1000 Gy for *A. ricciae* and *P. roseola* (Fig. 2B). A complete sterilization of isolated animals was achieved after a dose of 2000 Gy for *Habrotrocha* sp. *“Chile”* and *A. ricciae* species. Half of *P. roseola* samples exposed to 1500 Gy were unable to reproduce. A decrease in *Habrotrocha* sp. *“Belgium”* fertility was observed at doses of 1000 Gy and higher leading to a complete sterilization starting at 1000 Gy (Fig. 2B). The two Antarctic species (*A. editae* and *M. jankoi*) started to be strongly affected by radiation at a dose of 500 Gy and higher leading to a rapid sterilization of both species (Fig. 2B).

Fertility after radiation exposure for the seven desiccation-tolerant species, measured as SD_50_, failed to show any phylogenetic signal (Pagel’s lambda = 0.1, *p* = 0.9998; Blomberg’s *K* = 0.7, *p* = 0.3874) and was neither explained by survival capabilities nor by the frequency of desiccation of the habitat where the bdelloid originated (Additional file 3: Table S1).

We also studied whether the radioresistance may be affected by the hydration status of irradiated bdelloids, focusing only on two species, *A. editae* and *A. vaga*. The exposure of individuals up to 750 Gy similarly affected survival and fecundity rates, regardless of whether the animals were in the hydrated or desiccated state (Additional file 1: Fig. S4), suggesting that the reduced fertility after irradiation of *R. macrura* and *R. rotatoria* was not impacted by their hydrated status.

### Recovery of the genomic integrity post radiation

**Fig. 5:**
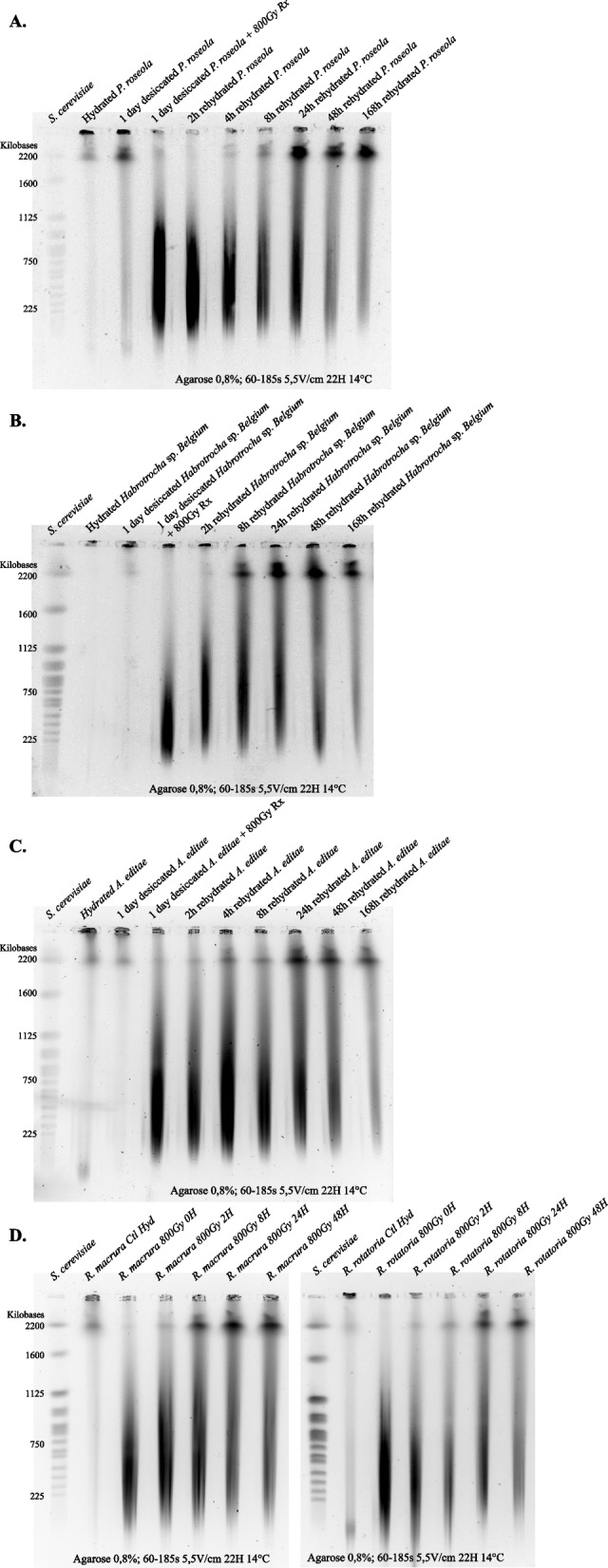
Genomic integrity of rehydrated *P. roseola* (**A**), *Habrotrocha* sp. *Belgium* (**B**), *A. editae* (**C**) after 1 day of desiccation with exposure to 800 Gy X-ray radiation and after 800 Gy X-radiation for hydrated *R. macrura* and *R. rotatoria* (**D**). Genomic integrity was assessed using pulsed-field gel electrophoresis (see Methods section) at different time points post irradiation (and rehydration for desiccation-resistant species). The first lane on the gels corresponds to the karyotype of *Saccharomyces cerevisiae*. The second lane corresponds to the control (1000 hydrated individuals, but 250 for *Rotaria* species). The third lane corresponds to 1-day desiccated bdelloids not submitted and submitted to 800 Gy X-ray radiation for A, B, and C, and to 250 hydrated individuals of *R. macrura* and *R. rotatoria* exposed to 800 Gy of X-ray for D. The other lanes correspond to 1000 desiccated individuals after 2, 4, 8, 24, 48, and 168 h of rehydration (**A**, **B**, **C**) or to 250 individuals after 2, 4, 8, 24, and 48 h post irradiation (**D**). The run parameters are documented under each gel

The ability of three selected desiccation-resistant species (*P. roseola*, *Habrotrocha* sp. “*Belgium”* and *A. editae*) and the two desiccation-sensitive (*R. macrura* and *R. rotatoria*) bdelloids to repair radiation-induced DNA DSBs was analyzed at different time points following 800 Gy of X-ray irradiation (Fig. 5). The genomic integrity of hydrated or 1-day desiccated bdelloid individuals is manifested by the presence of large DNA fragments that were retained in the gel plug and a faint signal at the upper limit of size separation of the gel (around 2200 kb), resulting from incidental DNA breakages arising from manipulations. After irradiation, the genomes of desiccation-resistant and desiccation-sensitive rotifer species were fragmented into pieces ranging from <225 to ~1000 kb (Fig. 5). During the recovery period under hydrated conditions, broken DNA fragments from the five different species reassembled to generate large DNA segments of ≥ 2200 kb, indicative of a DNA DSB repair mechanism acting in all bdelloid species. The repair mechanism did not completely restore the original unbroken pattern, with a smear of smaller fragments remaining after 48 or 168h post irradiation (Fig. 5) as observed previously for *A. vaga* species [20]. Similar conclusions could be drawn from PFGE analysis aimed at separating larger DNA fragments (Additional file 1: Fig. S5, see also Methods).

## Discussion

### Tolerance to desiccation and radiation are ancestral features of bdelloid rotifers

Bdelloids living in aquatic environments survive several generations without experiencing desiccation. Even those from temporary ponds may desiccate only once or a few times a year (e.g., *A. ricciae* from this study). In some limno-terrestrial habitats, desiccation frequency is much higher and may occur up to once a day, for example in lichens or mosses with humidity over the night and desiccation during the day. Thus, animals living in habitats experiencing frequent desiccation may desiccate several times in their life (i.e., lifespan = approx. 30 hydrated days), whereas animals living in permanent or temporary aquatic habitats almost never experience desiccation [28, 29]. In this study, we show that seven out of nine tested bdelloid species belonging to distinct families and originating from different environments (including both frequently and rarely desiccating habitats) are desiccation-tolerant, whereas *R. macrura* and *R. rotatoria*, with laboratory populations that were sampled in permanent water bodies, are not.

Although some variability between individuals might have been expected, no *R. macrura* or *R. rotatoria* individuals isolated from the field survived desiccation. The genus *Rotaria* belongs to the same family as *P. roseola* and *M. jankoi* (the Philodinidae family) that survived anhydrobiosis (Fig. 1). Within the *Rotaria* genus, some *Rotaria* species were previously described to be desiccation tolerant including the limno-terrestrial *R. sordida* and the aquatic *R. neptunoida* and *R. tardigrada* [13, 28]. The ancestral character reconstruction analysis performed here suggests that the common ancestor of bdelloid rotifers was desiccation resistant (scaled likelihood=0.99) and able to survive radiation (0.99). Our data therefore support the hypothesis that tolerance to desiccation is an ancestral feature of bdelloid rotifers, and consequently the evolution of radiation resistance, with a few species from the genus *Rotaria* probably having lost desiccation resistance secondarily when colonizing permanent water habitats. *Rotaria rotatoria* is known to be a complex of several species with rather different ecologies: organisms that can be identified with this species name are found in alpine acidic lakes, sewage treatment plants, marine waters, etc. making any general inference on the species unreliable [30]. The population we used in our study was unable to survive desiccation, as previously found [28]. *Philodina roseola* however, even though sampled from a permanent lake, was revealed to be resistant to desiccation: indeed, this species is known to live mostly in ephemeral water bodies with frequent desiccation [31], even if the population we used came from a permanent water body. The success of bdelloid rotifers to survive desiccation must be viewed as a combination of parameters including their own biological response but also external parameters independent of the rotifers. Several physical parameters were previously described to impact the recovery success of bdelloids exposed to desiccation (e.g., dehydration rate, relative humidity). Therefore, some variations of the data presented in this study may be expected with alternative desiccation assays. In the case of the two *Rotoria* species, alternative protocols were tested to check the potential resistance of these species to complete desiccation, all without success. Pursuing the characterization of other bdelloid species, with animals collected from different environmental conditions, may improve our capacity to decipher whether the ancestral bdelloid was most likely from a rarely (e.g., lake or pond) or a frequently desiccating habitat (e.g., moss, lichen, soil). Finally, about 1% of the described bdelloid species are known to live in marine habitats [32]. At this time, the ecology and tolerance to desiccation of these species have not been studied yet.

### Radioresistance of desiccation-tolerant species is independent of the desiccation frequency of their original habitat

By analogy with the desiccation- and radiation-resistant bacterium *Deinococcus radiodurans*, it was previously hypothesized that the radiation tolerance of bdelloid rotifers was a consequence of their evolutionary adaptation to semi-terrestrial environments, resulting from frequent cycles of desiccation and rehydration [17, 22]. In line with this hypothesis, Hespeels et al. [19] demonstrated that DNA DSBs do accumulate during prolonged desiccation in the bdelloid species *A. vaga*, a deleterious type of DNA damage that is also detected following exposure to IR [17, 20]. In congruence with these data, our study confirmed that for all the three tested desiccation-resistant species (*A. editae, P. roseola* and *Habrotrocha* sp. *“Belgium”*) DNA DSBs indeed accumulated in the genome as a function of time spent in dry state (Additional file 1: Fig. S1). This capacity to endure and repair massive genome breakage may have enabled both prokaryotic and eukaryotic species to also acquire radiation tolerance (Figs. 2, 3, and 5).

The survival to desiccation in bdelloids was correlated with higher survival among species experiencing frequent desiccation as it was expected from samples collected from moss, lichens, and dry grass of the Atacama Desert. *P. roseola* and *A. ricciae*, sampled respectively from permanent and temporary ponds, however retained the capacity for desiccation confirming the ancestral origin of the trait.

Radiation survival was high for all species, with the capacity to repair DNA DSBs being a potentially ancestral feature of the group (Fig. 5). Irradiation, up to 1500 or 2000 Gy has no effect on survivorship of desiccated bdelloids when compared to unirradiated animals. Even the desiccation-sensitive species were unaffected, in terms of survival in hydrated state, by doses up to 1000 Gy. In contrast, the fertility after radiation exposure was neither correlated to the frequency of desiccation of the habitat where the animals came from, nor to their survival capacity. The desiccation-sensitive species (*R. rotatoria* and *R. macrura*) are the most radio-sensitive ones with a SD50 <150 Gy (Figs. 3 and 4).

The Atacama Desert and the Antarctic continent host many extremophiles due to selection pressure applied by harsh environmental challenges such as exposure to long periods of desiccation or freezing, intense UV, high and low temperatures, or high-salt concentrations [33, 34]. As a consequence, it was not surprising to find microorganisms presenting a high resistance to UV radiation or ionizing radiation such as *Deinococcus* species in those samples [35–38]. However, how such habitats have been colonized by metazoans and how such environments have shaped desiccation and radioresistance remains poorly studied. This study provides the first record comparing desiccation and radiation resistance of three related species coming from environments as different as the Atacama Desert and Antarctic continent, respectively. At first glance, these three bdelloid species (namely *Habrothrocha* sp. *“Chile”* from the Atacama Desert, *M. jankoi* and *A. editae* from Antarctica) presented the highest survival rate after their exposure to a short period of desiccation (i.e., 24h) among the analyzed species (Fig. 2). In term of radioresistance, the survival rate of desiccated animals from the seven tested species exposed to a high dose of X-ray (≥1500Gy) was not significantly impacted in comparison with non-irradiated individuals (2). In a previous study, we reported that the survival rate of desiccated *A. vaga* individuals started to be affected after a dose of 5000 Gy with a complete extinction after exposure to 7500Gy. It remains unknown if this threshold is common to all bdelloid species or if some species can successfully challenge this limit. In contrast, exposure to radiation differentially affected the fertility of the tested bdelloid species and these data are not explained by survival capabilities or by their exposure to desiccation during their lifecycle. Indeed, *A. editae* and *M. jankoi* from Antarctica were characterized by the lowest SD_50_ (382±1 and 403±4 respectively) of all the tested desiccation-tolerant species, while *Habrotrocha* sp. *“Chile”* was in the range of maximal radioresistance (i.e., SD_50_ comprised between ~1000 and 1200 Gy) comparable to *A. vaga*, *A. ricciae*, and *P. roseola*. Selection pressure involved in the evolution of extreme desiccation resistance and radiotolerance in bdelloid rotifers is not limited to species colonizing extreme environments on Earth like the Atacama Desert or Antarctica, otherwise depicted as “Mars-like environments,” but also include limno-terrestrial environments such as lichens and mosses and permanent or temporary aquatic environments as reported for *P. roseola* and *A. ricciae*. As resistance to desiccation is not restricted to extreme environments, other factors may also be involved in the maintenance of such features among bdelloids. For example, anhydrobiotic capabilities of bdelloid rotifers have been previously described as a specific feature of bdelloid rotifers allowing them to escape from parasites present in their environment and involved in their ability to colonize new environments by wind dispersal [39].

The analyzed animals in this study were isolated from distant geographical areas characterized by different environmental parameters. Given the sharp differences in bioclimatic parameters between sampling areas, one could speculate that such contrasting parameters related to solar irradiance, UVA exposure, precipitation, and temperature may shape desiccation tolerance through evolutionary selection pressure. A preliminary analysis revealed that some bioclimatic variables (i.e., total irradiance, UVA exposure, level of precipitation, and maximum temperatures) may indeed have a significant effect on survival after radiation (Additional file 4). Such analyses remain to be expanded covering more species from additional bioclimatic areas before any reliable inference could be used to address convincing hypotheses on the effect of climate on desiccation and radiation resistance in bdelloids.

### Partial recovery of the genomic integrity following exposure to ionizing radiation in desiccation-tolerant and desiccation-sensitive bdelloid species

As previously reported for *A. vaga,* our data confirm that many bdelloid rotifers can recover from desiccation or remain active despite massive DNA damage induced by X-ray radiation (Figs. 2, 3, and 5). Interestingly, both the desiccation-tolerant and desiccation-sensitive bdelloid species are shown to survive high doses of ionizing radiation and activate their DNA repair machinery to reduce massive amounts of DNA DSBs caused by this genotoxic stress. The major part of DNA repair takes place within 24 h following irradiation and then reduces or stops, as was observed in Hespeels et al. [20] and Terwagne et al. [40], leaving incompletely reassembled chromosomes or a smear of fragments (Fig. 5) [20, 40].

Our data also shows that fertility is more sensitive to radiation than the ability of individuals to survive and to resume normal activities. This difference of radiotolerance is likely attributed to the nature of cells evaluated through survival or fecundity rate assays. Indeed, bdelloids are eutelic and in the adults their somatic cells are not engaged in any cell division or mitosis. The partial recovery of genomic integrity observed through PFGE reflects the somatic DNA repair in bdelloid individuals, which might be incomplete but sufficient to maintain gene expression for survival. In contrast, cells of the germline of bdelloid rotifers have to undergo active multiplication from the initial oocyte up to the end of development, requiring the full recovery of genomic integrity and therefore being more sensitive to the remaining damage induced by radiation [41, 42]. Therefore, it may be suggested that eutely is a key but not a sufficient character to explain the desiccation and radiation resistances (at survival level) among bdelloids.

Since oocytes and embryos likely represent a minority of cells in an adult population (i.e., ~20–40 oocytes *vs* ~1000 somatic nuclei [40, 43]), their contribution to the PFGE analysis performed on whole animal populations is presumably diluted by the pattern given by the somatic cells. From this analysis, it is therefore difficult to determine whether the DNA repair mechanism(s) acting in the somatic cells to ensure sustained cellular function and viability are distinct from the mechanism(s) acting in the germ line and embryos to ensure proper development, though the constraints imposed by DNA damage are clearly different in the two cell types. Terwagne et al [40] recently demonstrated that in the bdelloid rotifer *A. vaga* DNA repair in somatic nuclei occurs rapidly, producing a partially reassembled genome, while germline DNA repair is delayed and takes place during oogenesis, when the oocyte of *A. vaga* undergoes maturation through a non-reduced meiosis. Indeed, they observed homologous chromosome pairing in *A. vaga* during prophase I and the genomes of the descendants had the same restriction profiles in PFGE as the mother. Detecting a non-canonical meiosis essential for germline DNA repair in bdelloid rotifers provides the potential for new insights into their enigmatic long-term evolution. The exact nature of these repair mechanism(s) is currently being studied.

*R. macrura* and *R. rotatoria*, living in aquatic environments, were unable to survive desiccation and showed higher reproductive sensitivity to radiation than desiccation-resistant species (SD_50_ = 31 ± 1 Gy and 137 ± 7 Gy, respectively). It may be argued that differences reported from desiccation-resistant and desiccation-sensitive species could be linked with the desiccated or hydrated status of exposed animals. Indeed, the exposure of hydrated cells may be associated with higher level of cellular damages induced by water radiolysis [44]. However, this hypothesis was ruled out since we did not observe any difference in terms of survival and fertility for the desiccation-resistant species *A. editae* and *A. vaga* exposed to X-rays in desiccated and hydrated states (See Additional file 1: Fig. S4 and [20]). The higher radio-sensitivity expressed by *R. macrura* or *R. rotatoria* in terms of fertility might be linked to their viviparous mode of reproduction and the higher sensitivity of their embryos to these genotoxic stresses. An alternative hypothesis is that the observed pattern indeed reflects their differential resistance capabilities, and since these species inhabit permanent waterbodies, they might have lost secondary some critical physiological or molecular features to survive desiccation and radiation.

### Still so much to learn from bdelloids

The success of several organisms to deal with desiccation and radiation resistance is frequently linked to their powerful antioxidant systems that scavenge free radicals induced by these genotoxic challenges. The absence of an efficient antioxidant system or its saturation by high doses of radiation have been associated with an accumulation of damaged proteins leading to sterility and death, referred to as the “death by protein damage” hypothesis [45–49]. According to this hypothesis, the decrease and loss of fertility reported here for *R. macrura* or *R. rotatoria* following exposure to IR might be attributed to the progressive accumulation of protein damage and the inactivation of their DNA repair system preventing proper transmission of genomic material to the progeny [49, 50]. Nevertheless, no impact on the kinetics of DNA repair was observed in these bdelloid rotifer species exposed to 800 Gy of X-ray ruling out the hypothesis that the sterilization was associated with a complete inactivation of the DNA repair machinery. The variation in the rate of fertility following exposure to IR could be linked to their distinct reproductive physiology (ovi- and viviparous). It seems unlikely that the conservation of DNA repair proteins activity reported at the somatic level was lost among germinal cells. Nevertheless, our assay lacks the necessary resolution to define the degree of efficiency of the DNA repair machinery observed among bdelloids and its fidelity. Therefore, we cannot exclude that radiotolerance at the germinal level may be due to different efficiencies of complex DNA damage repair in different species. Future experiments, including for example single-cell transcriptomics, are required to highlight specific actors expressed at somatic and germinal cell levels and will contribute to a fine overview of desiccation/radiation tolerance of bdelloids. Finally, additional studies on radiation tolerance in bdelloid species adapted to permanently hydrated conditions, including marine species, are needed to obtain a more comprehensive picture of radiation tolerance in bdelloid rotifers.

While the ancestral state in bdelloid rotifers was apparently desiccation and radiation resistant, secondary changes in these resistances might have occurred following the changes in embryo development (oviparous to viviparous) and in habitat type (frequently desiccating limno-terrestrial to rarely desiccating aquatic habitats).

## Conclusions

Here, we investigated and compared nine bdelloid species collected from both mild and harsh environments (including individuals sampled among the Atacama Desert and Antarctica), addressing the correlation between the ability of bdelloid rotifers to survive desiccation and their capacity to repair massive DNA breakage in a phylogenetically explicit context. First, we confirmed that desiccation was associated with the loss of genomic integrity among bdelloids. Our analysis reveals that DNA repair prevails in somatic cells of both desiccation-tolerant and desiccation-sensitive bdelloid species. However, the fertility of two desiccation-sensitive species, *Rotaria macrura* and *Rotaria rotatoria*, was more severely impacted by low doses of radiation than that of desiccation-resistant species. Bdelloids isolated from harsh environments were not characterized by a higher radioresistance than species found in more temperate environments. Tolerance to desiccation and radiation are supported as ancestral features of bdelloid rotifers, with a few species having lost this trait when colonizing permanent water habitats. Together, our results highlight the importance to studying distinct species sampled from the wild within a specific clade to obtain a more comprehensive overview of the variation in their tolerance to extreme conditions. By studying this diversity, a better comprehension of the evolution of resistance to desiccation and radiation among bdelloids may appear.

## Methods

### Bdelloid rotifer cultures

The radioresistance of nine bdelloid species originating from various environments/origins around the world was investigated (See Fig. 1). Data for *Adineta vaga*, on a population originating from a moss in Italy, was gathered from previously published studies [19, 20]. *Adineta ricciae* was isolated from rehydrated sediment collected from a weedy temporary pond (billabong) close to Ryans Lagoon Nature Conservation Reserve, Australia (WGS84 coordinates: 36° 06′ 32.1″ S, 146° 58′ 37.5″ E), and kindly provided to us by C. Wilson [14, 51]. *Philodina roseola* was isolated from a permanent pond in Burlington, USA (36° 5′ 12.1992″ N, 79° 27′ 54.2736″ W), and provided by D. B. Mark Welch [52]. Both species have been cultivated in laboratory for at least 20 years. *Macrotrachela jankoi* and *Adineta editae* cultures were started from a single individual isolated from an Antarctic sample (65° 15′ 03.60″ S, 64° 14′ 33.50″ W) provided by Karel Janko and morphologically determined by N. Iakovenko [6]. *Habrotrocha* sp. *“Belgium”* was obtained by culturing a single individual collected by N. Debortoli from a lichen in Namur, Belgium (Debortoli et al., 2016) (50° 28′ 03.2″ N, 4° 51′ 25.9″ E). *Habrotrocha* sp. *“Chile”*, isolated from a dry plant sample in the Atacama Desert, Chile (23° 29′ 28.8″ S, 67° 46′ 09.4″ W), was collected and kept as a lab culture by J. W. Larrick. *Rotaria macrura* and *R. rotatoria* were collected from an artificial permanent pond in Belgium (50° 29′ 25.0″ N, 5° 02′ 20.8″ E) by A. Houtain. Experiments on *Rotaria* individuals were performed using a mix of individuals collected directly from the wild or temporarily maintained in the laboratory. All bdelloid cultures were maintained hydrated in Petri dishes supplemented with natural spring water (Spa®), in thermostatic chambers at 21°C and fed with sterilized lettuce (*Lactuca sativa*) juice.

### Phylogenetic analysis

The phylogenetic relationships between the tested species were inferred using fragments of two DNA markers: the mitochondrial Cytochrome C oxidase (COI) and the nuclear 18S rRNA gene following PCR amplification and DNA sequencing of 1 individual per clone [25, 53] (Additional file 3: Table S2). A relative time-calibrated phylogeny was reconstructed in BEAST version 2.6.0 [54], through the CIPRES portal [55], from the matrix of concatenated alignments; the analysis was run using uncorrelated parameters for each gene partition including the GTR + Γ + I substitution model, relaxed lognormal clock, constant birth rate prior, and a random starting tree. The MCMC chain was run for 100,000,000 generations and sampled every 10,000 generations; the first 20% of the trees were discarded as burnin to obtain an output tree with maximum clade credibility.

### Desiccation, X-ray exposure, survival rate

The desiccation protocol has been previously described and was already demonstrated to improve previously published protocols in terms of survival [19]. Briefly, cultures were washed with 15 mL Spa® water the day before their collection. Individuals were detached from the Petri dish with the addition of 450 μL of NaCl 5 M per plate followed by vortexing. Animals were transferred to a 15-mL Falcon tube for centrifugation. Pellets were resuspended in 0.5 mL of Spa® water and placed at the center of Petri dishes containing 30 mL of 3% Low Melting Point agarose (LMP agarose, Invitrogen). LMP agarose plates containing hydrated individuals were placed in a climatic chamber (WEKK 0028) for dehydration with the following parameters: (1) linear decrease in relative humidity (RH) from 70 to 55% for 17 h (T° 21–23°C), (2) linear decrease in relative humidity from 55 to 41% for 1 h (T° 21–23°C), and (3) maintenance at 41% RH and 21–23°C for the desiccated period (from 1 to 30 days). The number of animals used for desiccation assays depended on the growth rate of the cultures of each bdelloid species. The minimum number of animals was 8000, 15,000, 20,000, 27,000, 30,000, and 60,000 for *M. jankoi*, *Habrotrocha* sp. *“Belgium”*, *P. roseola*, *Habrotrocha* sp. *“Chile”, A. editae,* and *A. ricciae*, respectively. During dehydration, desiccation-resistant species formed groups/clusters of desiccated rotifers. As clustering of individuals is positively linked to their survival rate (see Hespeels et al. [19]), this study only focused on clustered rotifers. A minimum of three replicates were tested for each species (exception for: *A. editae* 1500 Gy n=2 and 2000 Gy *n*=1; *A. ricciae* 100 Gy *n*=2 and 2000 Gy *n*=2). Due to slow growth of *R. macrura* and *R. rotatoria* under laboratory conditions, we did not obtain clonal cultures of >500 individuals for these species. Therefore, desiccation tolerance was evaluated on ~500 *R. macrura* individuals isolated from the field using the desiccation protocol described above. For *R. macrura*, two trials of two biological replicates each were performed from samples isolated in June 2018 and November 2018. For *R. rotatoria*, the desiccation tolerance was initially evaluated on ~1000 individuals isolated from the field (February 2021). Later (March 2021), three desiccation assays were performed using 3000, 10,000, and 25,250 individuals originated from a polyclonal culture maintained in laboratory for 2 months. The full dataset can be found in Additional file 3.

Desiccated bdelloids were irradiated with 225 kVp X-rays at a dose rate of ~7.8 Gy/min (using X-ray irradiator PXi X-RAD 225 XL). Increasing irradiation times were used to reach final doses. Samples were maintained in a refrigerated water bag to mitigate heating due to X-rays. Desiccation-tolerant species were irradiated in a desiccated state up to 1500 Gy (for *Habrotrocha* sp. *“Belgium”* and *P. roseola*) and 2000 Gy (for *A. editae*, *A. ricciae, M. jankoi* and *Habrotrocha* sp. *“Chile”*), respectively. The radio tolerance of the desiccation-sensitive *R. macrura* and *R. rotatoria* species was, however, evaluated on hydrated animals exposed to 1000 Gy in a final volume of 10 mL. In order to check the effect of hydration on irradiation, hydrated *A. editae* individuals were also exposed up to 750 Gy. As reported for desiccated samples, hydrated specimens were stored in a refrigerated water bag during the irradiation process.

After desiccation, and after irradiation, dried bdelloid individuals were rehydrated using 15 mL Spa® water and stored at 21°C for 48 h. Bdelloids were considered alive when they had fully recovered motility or when the mastax moved in contracted individuals. All living and dead animals were collected separately using a micropipette. Then, the survival rate was estimated manually by counting living and dead individuals under the binocular stereoscope. When the number of individuals was greater than ~500, the number of individuals was extrapolated by counting 6 times the number of living/dead animals observed in 2 μL of a final volume of 2–5 mL (raw data in Additional file 2).

For the survival rate of hydrated and optionally irradiated *R. rotatoria* and *R. macrura*, 60 individuals were isolated from control and irradiated culture plate. The isolated rotifers were deposited individually in a well of a 12-well Petri plate. Then, wells were filled with 2 mL of Spa® water and 50 μL of sterilized lettuce juice. After 48h, the survival rate was recorded by observation of active or dead individuals under a binocular stereoscope.

### Fertility assays

The reproductive capacity of bdelloid species was defined as the ability of each individual to lay eggs (or newborns for viviparous species) and to develop clonal populations following desiccation and irradiation. We tested the fertility of 1-day desiccated and irradiated individuals by randomly selecting and isolating a minimum of 60 successfully rehydrated individuals per condition. Each isolated female was deposited in a well of a 12-well Petri plate. Each well was filled with 2 mL of Spa® water and 50 μL of sterilized lettuce juice. After 30 days, for all oviparous species, wells were observed under a binocular stereoscope checking for: (1) the presence of a population (minimum 2 adults and 1 egg per well), (2) the presence of only eggs that did not hatch (+ the starting adult defined as a sterile individual), and (3) the presence of only dead individual(s). *R. macrura* and *R. rotatoria* are desiccation-sensitive viviparous rotifers, thus, they do not produce eggs but only fully developed newborns. For these viviparous species, fertility after irradiation was determined as follows: 60 animals were isolated in a well of a 12-well petri plate filled with 2 mL of Spa® water and 50 μL of sterilized lettuce juice. The number of animals (alive or not) found in each well was counted after 30 days. Effective reproduction thus was calculated.

Based on these data, we evaluated the minimal radiation dose required to sterilize 50% of the population. A similar approach to the evaluation of a standard LD_50_ was applied to define the Sterilizing Dose 50 (SD_50_). All curve fittings were performed with the OriginLab® software (Northhampton, MA, USA). A dose-response equation was used to fit fertility data (Eq. 1):

1 $$Fertility={A}_1+\frac{A_2-{A}_1}{1+{10}^{\left(\mathit{\log}{x}_0-x\right)p}}$$

where A_1_ is the bottom asymptote *(*i.e., *representing the fertility of sterilized animals),* A_2_ is the top asymptote (i.e., *representing the fertility of control animals)*, logx_0_ correspond to SD_50_, *p* is the hill slope, and *x* the dose of irradiation.

### Statistical analyses

We addressed whether the degree of susceptibility to desiccation and to radiation for each species was related to the desiccation frequency of the original habitat where the population was sampled in the field, namely frequently desiccating (limno-terrestrial habitats as in the water films surrounding particles in terrestrial habitats like mosses, lichens, soil), where desiccation is experienced by each animal several times during its life) or rarely desiccating (aquatic water bodies like permanent or temporary ponds), where desiccation is almost never experienced or only once every several generations of life that is usually in hydrated conditions. Information on the habitat for each species, either frequently or rarely desiccating, was gathered from the population of origin and compared with what is known from the literature for the species (e.g., Donner [31]). The degree of susceptibility to desiccation for each species was measured as the proportion of surviving animals after the experiment, averaged across the replicates.

We started analyzing the correlates of desiccation for all the nine species, and then tested whether radiation survival and fertility for the seven desiccation-tolerant species could be explained by their desiccation capabilities. Survival and fertility after radiation was explicitly included in the analysis only for species that could desiccate and produce desiccated stages for the experiments, in order to avoid any bias due to the presence of water (possibly linked with a higher level of ROS production during exposure) or by the metabolic activity during radiation exposure. As a metric of fertility after radiation, we used SD_50_, however only for exposure while desiccated. We assessed the correlation between values of survival at 500, 1000, and 1500 Gy using Pearson’s correlation tests [56].

The analyses were performed controlling for the phylogenetic non-independence of the species used in the analyses by performing phylogenetic generalized least squares (PGLS) in R 4.0.3 (R Core Team, [57]) using package caper v1.0.1 [58]. As a phylogeny for the PGLS, we used the ultrametric tree obtained from BEAST on the combined alignment of COI and 18S datasets, with branch length transformations (lambda, delta, and kappa) optimized by maximum likelihood given the data and the model [59]. Before performing PGLS, we checked whether there was a phylogenetic signal in the degree of susceptibility to desiccation and survival and fecundity after radiation. Phylogenetic signal was assessed through Pagel’s lambda and Bloomberg’s K in the R package phytools v0.7.20 [60].

We also performed ancestral character estimation of each of the analyzed metrics, in order to identify whether some features were already present and then lost in some species, or if they evolved only in a few clades from a common ancestor without such feature. Such estimation was performed in the R package ape v5.3 [61].

Before performing analyses on survival and reproductive capacity to desiccation and to radiation, we assessed whether different levels of treatment resulted in significantly different responses in survival compared to the controls, between the different treatments, and between species as performed in [20]. Effects of radiation dose were analyzed using a generalized linear model (using a Gaussian family for survival and reproduction rates, or a Poisson family for individual count), followed by Tukey’s test for post hoc multiple comparisons. This approach did not restrict the statistical analysis to the comparison of each condition versus the control condition (i.e., non-irradiated samples), but also between each condition. Multiple comparisons between each dose ensured to discriminate significant differences between all conditions. Statistical analyses were performed with the aid of the car v3.0-7 [62] and multcomp v1.4-13 packages [63].

### Genomic DNA integrity

Accumulation of DNA double-strand breaks (DSBs) and genome integrity of hydrated/desiccated/irradiated bdelloids were examined by pulsed-field gel electrophoresis (PFGE) using a protocol adapted from [19]. For the desiccation-tolerant species, one species from each of the three bdelloid families was included in the analysis (*A. editae* for Adinetidae, *P. roseola* for Philodinidae, and *Habrotrocha* sp. *“Belgium”* for Habrotrochidae) and investigated for the accumulation of DNA DSBs on hydrated and desiccated samples, exposed to 1, 14, and 30 days of dry period.

For the same three species, the kinetics of DNA DSB repair was examined on early desiccated animals (i.e., after 1 day of desiccation) exposed to 800-Gy X-ray radiation. Genomic integrity was examined after 0, 2, 4, 8, 24, 48, and 168 h post irradiation. In addition, the DNA repair kinetics of the desiccation-sensitive species, *R. macrura* and *R. rotatoria* (family Philodinidae), were evaluated on hydrated populations after 0, 2, 8, 24, and 48h post irradiation. For these two species, 250 individuals were isolated from the wild for each condition and irradiated in a final 250 μL water volume. For each time point, samples were pelleted by centrifugation (2 min 16,000*g*) in 1.3 ml of EDTA 50 mM, Tris HCl 10 mM, pH 8 and stored at −80°C. Agarose plugs were prepared by mixing 31 μL of EDTA 50 mM, Tris HCl 10 mM, pH 8 containing ~1000 individuals for most species and 250 individuals for the two *Rotaria* species due to a lower number of available animals, and 19 μL of melted CleanCut agarose 2% (BioRad, Hercules, CA, USA). After 15 min at 4°C, plugs were individually transferred into 500 μL of digestion buffer 100 mM EDTA, 50 mM Tris pH 8, supplemented with 1 mg/mL proteinase K (Thermo Fisher Scientific, Waltham, MA, USA) and 3.3% N-Lauroylsarcosine sodium solution. Plugs were stored for 1 h at 4°C and incubated for 18 h at 56°C. Then, plugs were rinsed three times with 0.5× Tris Borate EDTA (TBE) and kept for 3 h in 0.5× TBE at 4 °C. Finally, plugs were stored at 4°C in 500 μL 0.5 M EDTA pH 8 after a last rinse with 0.5× TBE before use. Plugs were loaded in a 0.8% agarose gel (Lonza, Rockland, ME, USA), and PFGE was performed on a BIORAD CHEF Mapper XA according to the following parameters: migration time = 22 h, temperature = 14 °C, volts/cm = 5.5, switch angle = 120°, switch times = 60 to 185 s with a linear ramp (resolution ranging from 225 to 1600 kb). In order to resolve larger DNA fragments (resolution between 2000 to 7000 kb), the following parameters were used: migration time = 96 h, temperature = 14 °C, volts/cm = 1.5, switch angle = 106°, switch times = 600 to 4800 s with a linear ramp. *Saccharomyces cerevisiae* and *Hansenula wingei* chromosomes were used as a size ladder depending on the resolution (BioRad, Hercules, CA, USA). Gels were stained with SYBR Gold (Invitrogen, Carlsbad, CA, USA) and analyzed with a BioRad Chemidoc XRS camera. Images were processed using ImageLab 3.0 and ImageJ. DNA DSBs induced by desiccation or radiation are expected to result in a loss of genomic integrity. Intact or slightly degraded chromosomes are expected to remain in the well. Indeed, assembled genomes of *A. ricciae, A. vaga,* and *R. macrura* were described to be ~ 174.5 Mb (12 chromosomes), 217 Mb (12 chromosomes), and 234.7 Mb [14] respectively. DNA segments migrating within the resolution size of the PFGE (~< 2.2 Mb or ~< 7 Mb) result from chromosome breakage, in agreement with recent guidelines [64].

## Supplementary Information

**Additional file 1: Figure S1.** Genome integrity of hydrated and desiccated bdelloids respectively *P. roseola*, *H. sp “Belgium”*, *A. editae*: PFGE (A) and Photometric Scans (B). The two panels show pulsed-field gel electrophoresis patterns (PFGE) obtained for 1000 bdelloids individuals that were kept hydrated or submitted to 1, 14 and 30 days of desiccation as indicated. Migration parameters are documented under each gel. Chromosomes of *S. cerevisiae* are used as size markers. Photometric scans were generated using ImageJ (see “Methods” section). Top flat lines from photometric scans correspond to saturated levels. **Figure S2.** Phylogenetic tree with mapped ancestral characters reconstructions (ACE) for desiccation (A) and habitat desiccation feature (B). Color codes are white = desiccation-sensitive and gray = desiccation-tolerant for A; light blue = rarely desiccating, dark green = frequently experiencing desiccation for B. **Figure S3.** Evaluation of Sterilizing Dose 50 (SD50, Gy) for various bdelloid species exposed to X-ray. For each replicate, 60 individuals were randomly isolated and individually placed in multiwell plates. Reproduction was evaluated by direct observation under binocular 30 days after irradiation and rehydration. All curve fittings were performed with the OriginLab® software (MA, United States). **Figure S4.** Comparison between the survival rate (A) and the reproductive capacity (B) of desiccated (light color) and hydrated (dark color) *A. editae* (color green) or *A. vaga* (color gray) individuals exposed to X-ray. Survival rate was evaluated 2 days post rehydration or post radiation on minimum of 3 replicates. The reproductive capacity was evaluated with a minimum of 3 replicates per dose (see Methods). For each replicate, reproduction was evaluated by direct observation under binocular 30 days after rehydration/radiation. Effective reproduction was validated when at least 2 adults and 1 egg were observed per well. Statistical analysis includes comparison between and within each group of desiccated and hydrated individuals. Data were visualized as dotplot (● = average value with plot of Standard Deviation). Group characterized letters indicate the significant differences between groups: a significant difference (Tukey test p-value <0.05) between two conditions is observed when these conditions do not share any letter. Data for *A. vaga* were adapted from Hespeels et al., [20]. **Figure S5.** Repair kinetic of rehydrated *A. editae* (A) *Habrotrocha* sp *Belgium* (B) and *P. roseola (*C*)* after 1 day of desiccation with exposure to 800 Gy X-ray radiation. The first lane on the pulsed-field gel electrophoresis correspond to the karyotype of *Saccharomyces cerevisiae (A and B.) or H. wingei (C)*. Second lanes correspond to the control (1000 hydrated individuals). Third lanes of Fig. 3 A/B correspond respectively to 1 day desiccated bdelloids not submitted and submitted to 800 Gy X-ray radiation. Other lanes correspond to 1000 desiccated individuals after 2, 4, 8, 24, 48 and 168 h of rehydration. The run parameters are documented under each gel.

**Additional file 2.** Survival and fertility raw data.

**Additional file 3: Table S1.** Output of the Phylogenetic Generalized Least Square (PGLS) model for effects on fecundity, measured as SD_50_, of habitat, desiccation, and radiation for the seven desiccation-tolerant species (adjusted R^2^ = 0.21 for A and 0.94 for B). The tables report the four predictors with estimates ± standard errors, t values and p values from the PGLS models. **Table S2.** GenBank Accession numbers of the sequences used for the study of the phylogenetic relationships. Data for *A. ricciae*, *A. vaga*, and *P. roseola* were obtained from the literature on previously published DNA sequence data for the lab cultures we used.

**Additional file 4.** Set of tests on potential correlates of survival capabilities, addressing whether the climatic conditions of the original habitat of each species could explain their susceptibility to desiccation or to radiation.

## Data Availability

All data generated or analyzed during this study are included in this published article [and its supplementary information files].

## Abbreviations

DSB: Double-strand breaks
GLM: Generalized linear model
HGT: Horizontal gene transfer
IR: Ionizing radiation (IR)
P: *P* values
PFGE: Pulsed-field gel electrophoresis
PGLS: Phylogenetic generalized least squares
