# Supplementary material for: Back to the roots, desiccation and radiation resistances are ancestral characters in bdelloid rotifers
Source: BMC Biol. 2023 Apr 7;21:72. doi: 10.1186/s12915-023-01554-w (PMC10080820; doi:10.1186/s12915-023-01554-w)
Supplement: Supplementary file 1 — Additional file 1: Figure S1. Genome integrity of hydrated and desiccated bdelloids respectively P. roseola, H. sp “Belgium”, A. editae: PFGE (A) and Photometric Scans (B). The two panels show pulsed-field gel electrophoresis patterns (PFGE) obtained for 1000 bdelloids individuals that were kept hydrated or submitted to 1, 14 and 30 days of desiccation as indicated. Migration parameters are documented under each gel. Chromosomes of S. cerevisiae are used as size markers. Photometric scans were generated using ImageJ (see “Methods” section). Top flat lines from photometric scans correspond to saturated levels. Figure S2. Phylogenetic tree with mapped ancestral characters reconstructions (ACE) for desiccation (A) and habitat desiccation feature (B). Color codes are white = desiccation-sensitive and gray = desiccation-tolerant for A; light blue = rarely desiccating, dark green = frequently experiencing desiccation for B. Figure S3. Evaluation of Sterilizing Dose 50 (SD50, Gy) for various bdelloid species exposed to X-ray. For each replicate, 60 individuals were randomly isolated and individually placed in multiwell plates. Reproduction was evaluated by direct observation under binocular 30 days after irradiation and rehydration. All curve fittings were performed with the OriginLab® software (MA, United States). Figure S4. Comparison between the survival rate (A) and the reproductive capacity (B) of desiccated (light color) and hydrated (dark color) A. editae (color green) or A. vaga (color gray) individuals exposed to X-ray. Survival rate was evaluated 2 days post rehydration or post radiation on minimum of 3 replicates. The reproductive capacity was evaluated with a minimum of 3 replicates per dose (see Methods). For each replicate, reproduction was evaluated by direct observation under binocular 30 days after rehydration/radiation. Effective reproduction was validated when at least 2 adults and 1 egg were observed per well. Statistical analysis includes comparison be [file 12915_2023_1554_MOESM1_ESM.docx]

Back to the roots, desiccation and radiation resistances are ancestral characters in bdelloid rotifers.

Figures S1-S5

**S1 Figure.** Genome integrity of hydrated and desiccated bdelloids respectively *P. roseola*, *H. sp “Belgium”*, *A. editae*: PFGE (A) and Photometric Scans (B). The two panels show pulsed‐field gel electrophoresis patterns (PFGE) obtained for 1000 bdelloids individuals that were kept hydrated or submitted to 1, 14 and 30 days of desiccation as indicated. Migration parameters are documented under each gel. Chromosomes of *S. cerevisiae* are used as size markers. Photometric scans were generated using ImageJ (see “Materials and Methods” section). Top flat lines from photometric scans correspond to saturated levels.

**
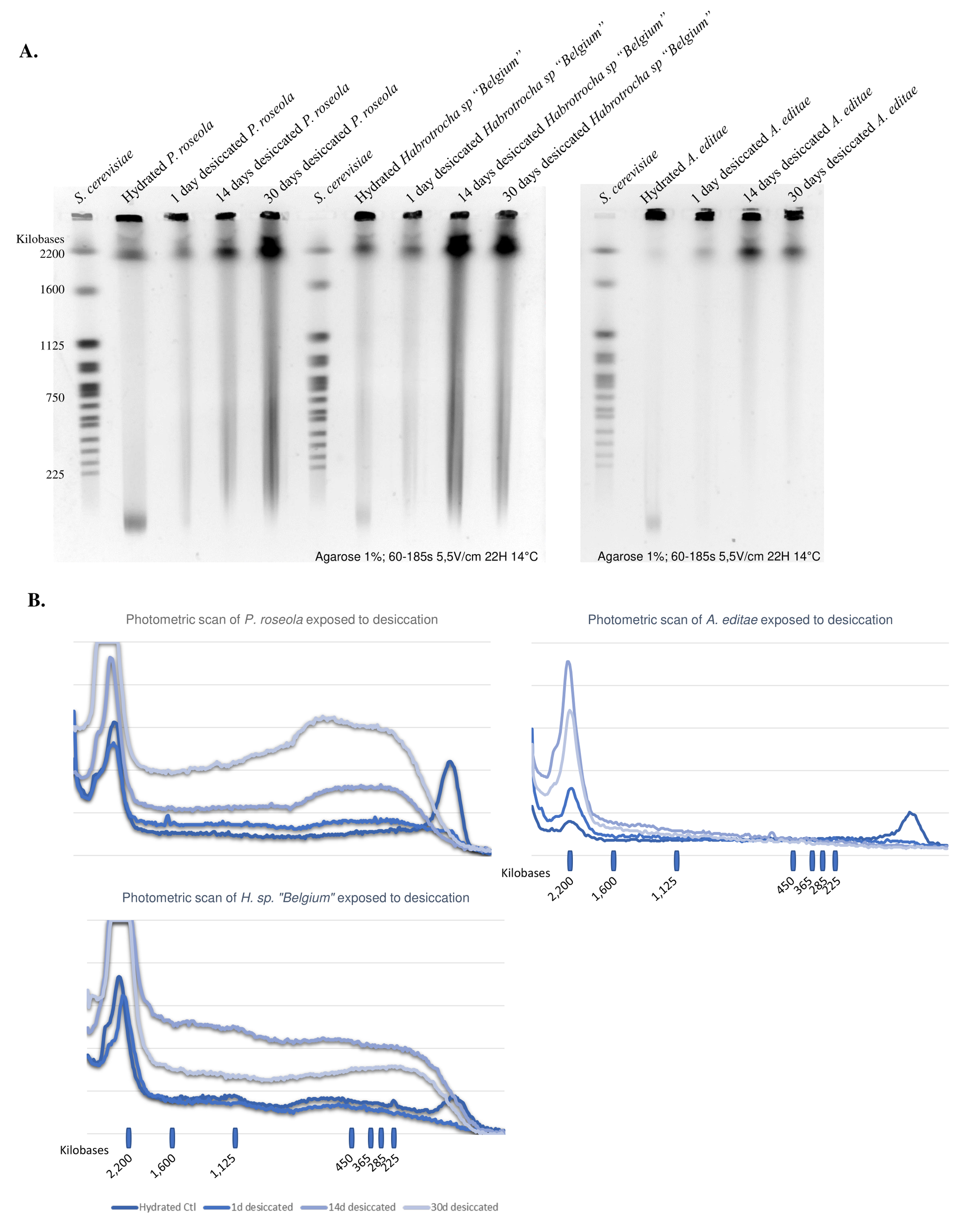
**

**S2 Figure.** Phylogenetic tree with mapped ancestral characters reconstructions (ACE) for desiccation (A) and habitat desiccation feature (B). Color codes are white = desiccation-sensitive and grey = desiccation-tolerant for A; light blue = rarely desiccating, dark green = frequently experiencing desiccation for B.

**
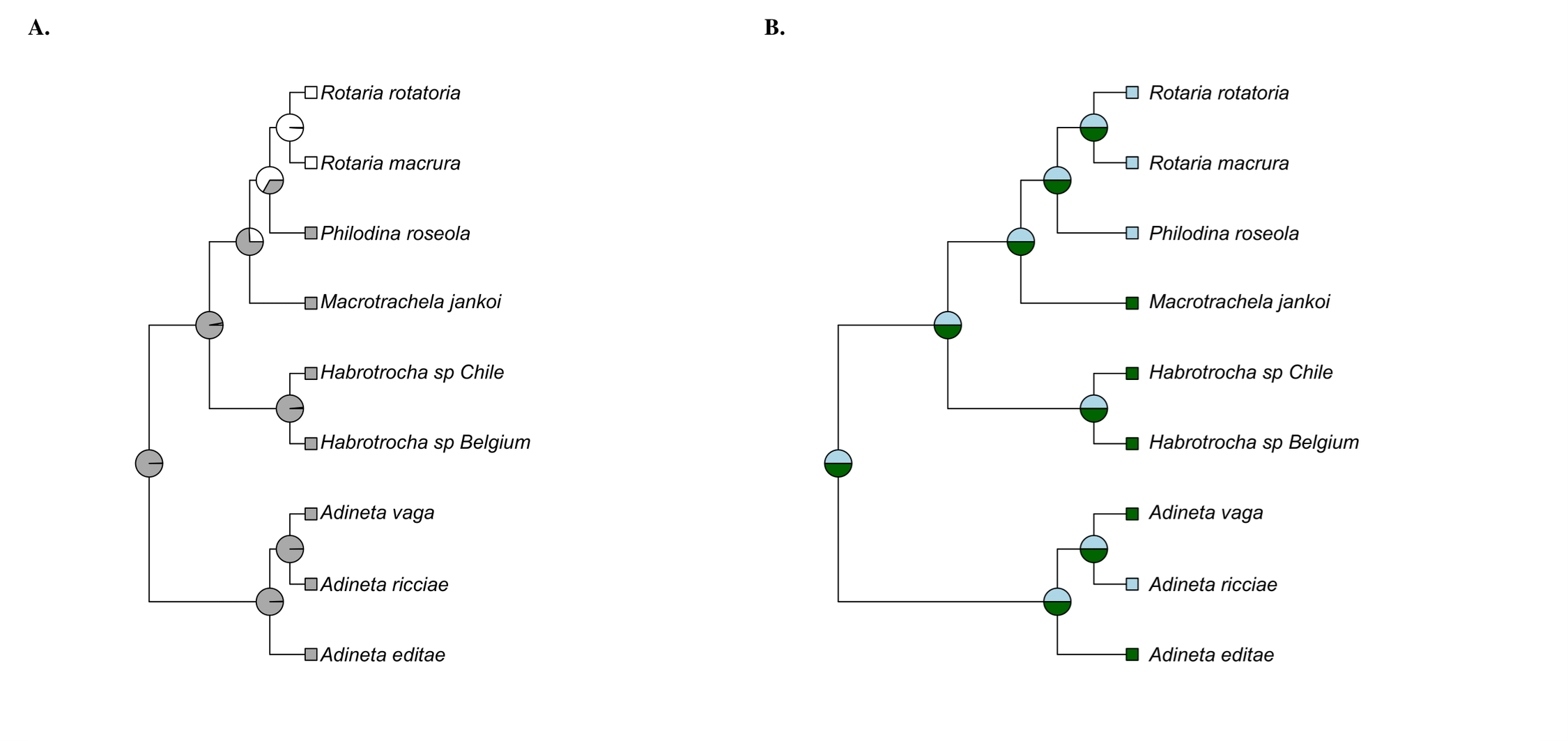
**

**S3 Figure.** Evaluation of Sterilizing Dose 50 (SD50, Gy) for various bdelloid species exposed to X-ray. For each replicate, 60 individuals were randomly isolated and individually placed in multiwell plates. Reproduction was evaluated by direct observation under binocular 30 days after irradiation and rehydration. All curve fittings were performed with the OriginLab® software (MA, United States).

**
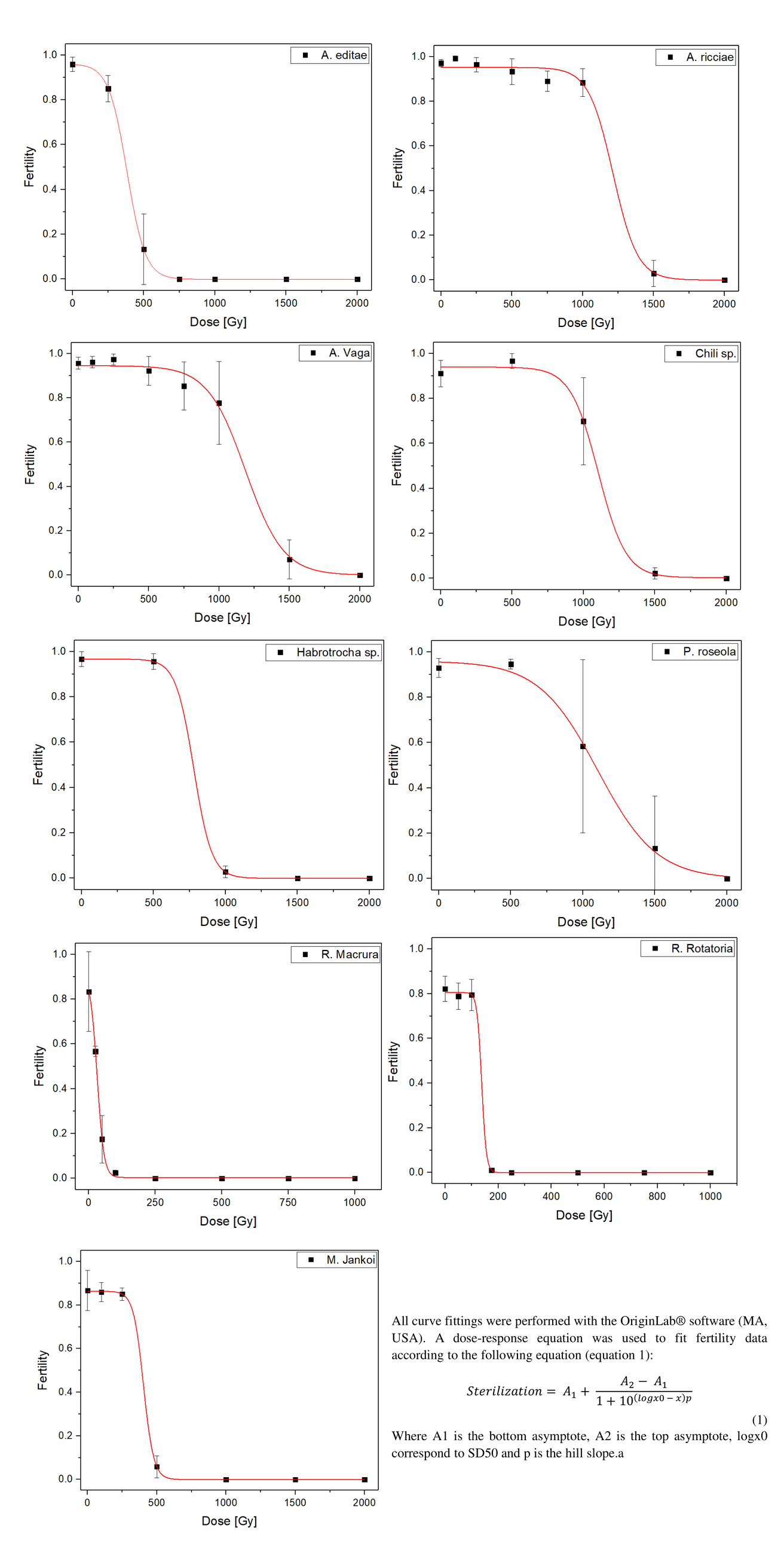
**

**S4 Figure.** Comparison between the survival rate (A) and the reproductive capacity (B) of desiccated (light color) and hydrated (dark color) *A. editae* (color green) or *A. vaga* (color grey) individuals exposed to X-ray. Survival rate was evaluated 2 days post rehydration or post radiation on minimum of 3 replicates. The reproductive capacity was evaluated with a minimum of 3 replicates per dose (See M&M). For each replicate, reproduction was evaluated by direct observation under binocular 30 days after rehydration/radiation. Effective reproduction was validated when at least 2 adults and 1 egg were observed per well. Statistical analysis includes comparison between and within each group of desiccated and hydrated individuals. Data were visualized as dotplot (● = average value with plot of Standard Deviation). Group characterized letters indicate the significant differences between groups: a significant difference (Tukey test p-value <0.05) between two conditions is observed when these conditions do not share any letter. Data for *A. vaga* were adapted from Hespeels et al., 2020

**
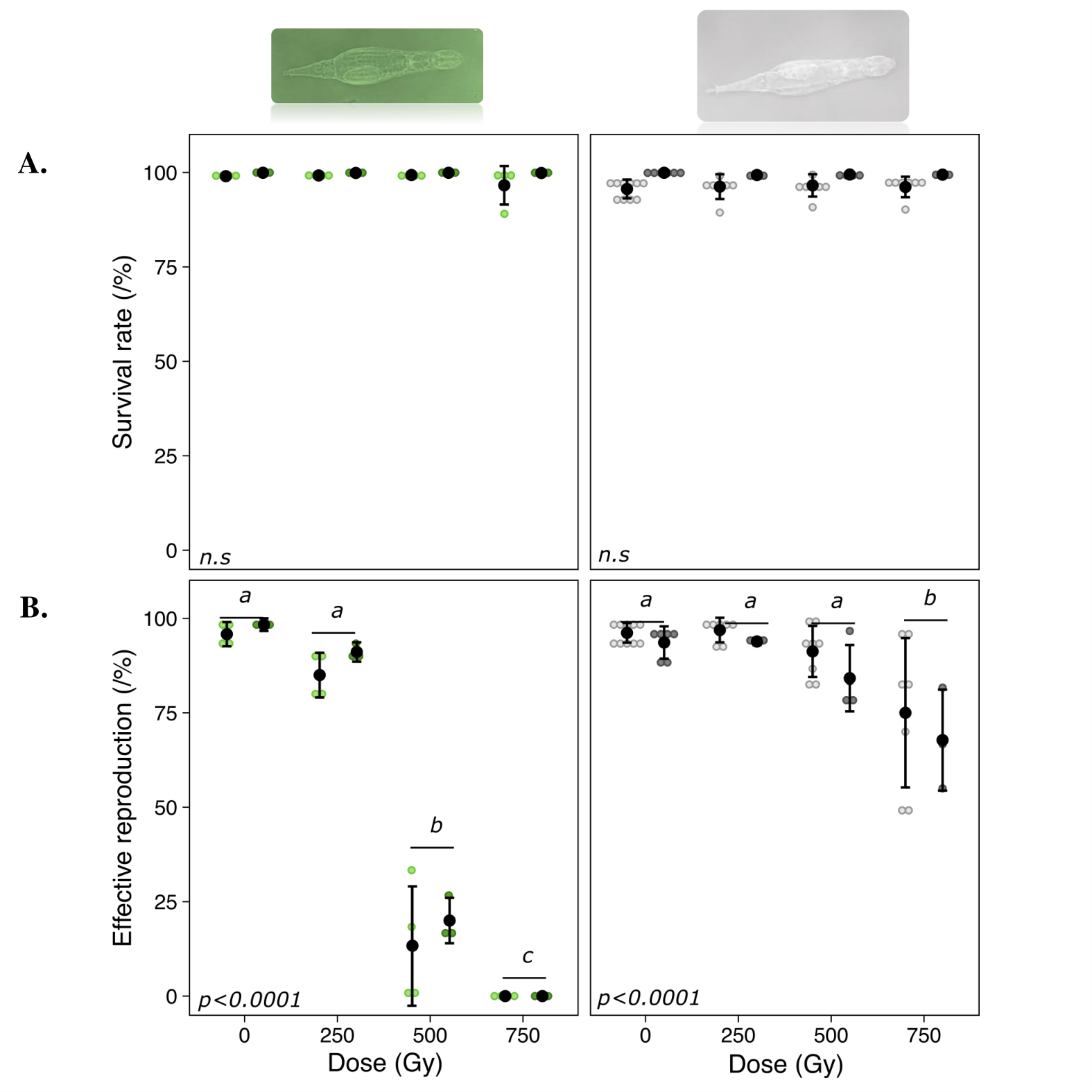
**

**S5 Figure.** Repair kinetic of rehydrated *A. editae* (A) *Habrotrocha* sp *Belgium* (B) and *P. roseola (*C*)* after 1 day of desiccation with exposure to 800 Gy X-ray radiation. The first lane on the pulsed-field gel electrophoresis correspond to the karyotype of *Saccharomyces cerevisiae (A and B.) or H. wingei (C)*. Second lanes correspond to the control (1000 hydrated individuals). Third lanes of Figure 3 A/B correspond respectively to 1 day desiccated bdelloids not submitted and submitted to 800 Gy X-ray radiation. Other lanes correspond to 1000 desiccated individuals after 2, 4, 8, 24, 48 and 168 h of rehydration. The run parameters are documented under each gel.

**
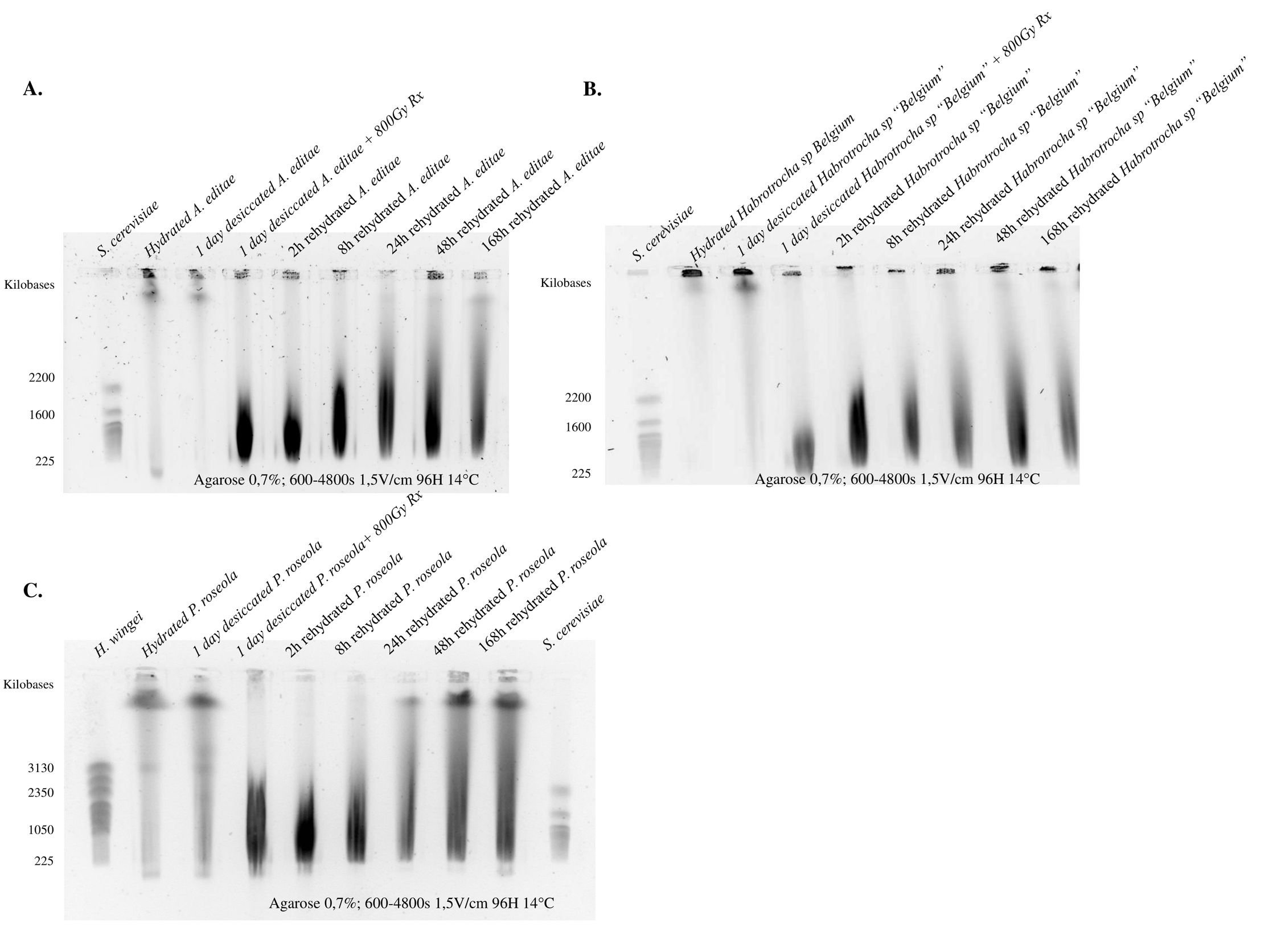
**
