# Supplementary material for: Back to the roots, desiccation and radiation resistances are ancestral characters in bdelloid rotifers
Source: BMC Biol. 2023 Apr 7;21:72. doi: 10.1186/s12915-023-01554-w (PMC10080820; doi:10.1186/s12915-023-01554-w)
Supplement: Supplementary file 3 — Additional file 3: Table S1. Output of the Phylogenetic Generalized Least Square (PGLS) model for effects on fecundity, measured as SD50, of habitat, desiccation, and radiation for the seven desiccation-tolerant species (adjusted R2 = 0.21 for A and 0.94 for B). The tables report the four predictors with estimates ± standard errors, t values and p values from the PGLS models. Table S2. GenBank Accession numbers of the sequences used for the study of the phylogenetic relationships. Data for A. ricciae, A. vaga, and P. roseola were obtained from the literature on previously published DNA sequence data for the lab cultures we used. [file 12915_2023_1554_MOESM3_ESM.docx]

Back to the roots, desiccation and radiation resistances are ancestral characters in bdelloid rotifers.

Table S1-S2

**Table S1.** Output of the Phylogenetic Generalized Least Square (PGLS) model for effects on fecundity, measured as SD_50_, of habitat, desiccation, and radiation for the seven desiccation-tolerant species (adjusted R^2^ = 0.21 for A and 0.94 for B). The tables report the four predictors with estimates ± standard errors, t values and p values from the PGLS models.

|  | predictor | estimate | t | p |
| --- | --- | --- | --- | --- |
|  | (intercept) | 10714.8 ± 5645.8 | 1.9 | 0.1540 |
|  | habitat | 1156.2 ± 887.6 | 1.3 | 0.2837 |
|  | desiccation | -224.3 ± 123.6 | -1.8 | 0.1672 |
|  | radiation | 11148.3 ± 6292.2 | 1.8 | 0.1746 |

**Table S2.** GenBank Accession numbers of the sequences used for the study of the phylogenetic relationships. Data for *A. ricciae*, *A. vaga*, and *P. roseola* were obtained from the literature on previously published DNA sequence data for the lab cultures we used.

| species | 18S | COI |
| --- | --- | --- |
| *Adineta editae* | OP331323 | OP320365 |
| *Adineta ricciae* | KM043251 | EF173188 |
| *Adineta vaga* | ASM2161353v1 | ASM2161353v1 |
| *Habotrocha* sp. *“Belgium”* | NA | OP320367 |
| *Habotrocha* sp. *“Chile”* | OP331324 | OP320366 |
| *Macrotrachela jankoi* | OP331325 | OP320368 |
| *Philodina roseola* | AF154567 | EF650520 |
| *Rotaria macrura* | OP331326 | OP320369 |
| *Rotaria rotatoria* | OP331327 | OP320370 |
